# Supplementary material for: “Energetics of the outer retina I: Estimates of nutrient exchange and ATP generation”
Source: PLoS One. 2024 Dec 31;19(12):e0312260. doi: 10.1371/journal.pone.0312260 (PMC11687866; doi:10.1371/journal.pone.0312260)
Supplement: S1 File — (DOCX) [file pone.0312260.s001.docx]

**Supporting Information**

**“Energetics of the outer retina I: Estimates of nutrient exchange and ATP generation.”**

**Authors:** Stella Prins, Christina Kiel, Alexander JE Foss, Moussa A Zouache, Philip J Luthert

Content

Supplementary Figures S1 to S10

Supplementary Tables S1 to S6

**Figure S1.** Summary diagrams. A: This diagram shows how the outer retinal circulation in the choroid supports RPE and inner segments and the inner retinal circulation, as well as supporting synapses in the outer plexiform layer, provides some support to the inner segments as well. B: This figure summarises the compartments captured by the flux balance model. The separate rod and cone models are individually coupled to the RPE. (Exchange between rods and cones is not included.) The dominant exchange with the circulation is with the choriocapillaris but a component of supply from the retinal circulation is included.


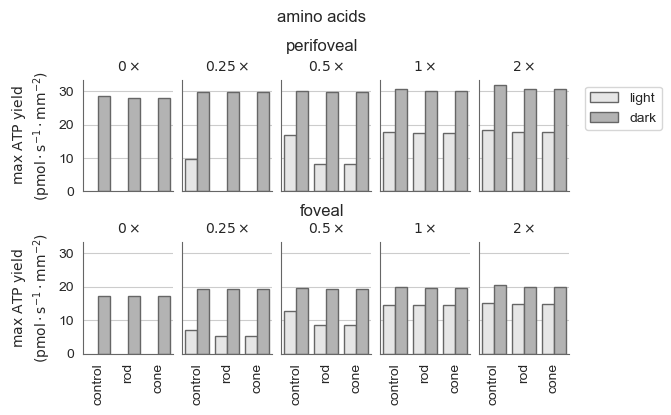
**Figure S2.** Maximal ATP hydrolysis rate with varying levels of amino acid influx rates. The maximal rate of ATP synthesis in pmol·s^−1^·mm^−2^ during light (light grey) and dark (dark grey), in absence (0 ×) and presence of different levels of amino acid influx (0.25 to 2 ×) of amino acids for the control model and for the retina-specific models. The control model contains two coupled Human1 models, while the retina-specific models consist of a RPE model coupled to a photoreceptor rod (‘rod’) or cone (‘cone’) model.

**Figure S3.** Maximal foveal ATP yield with increasing glucose influx. ATP hydrolysis was optimised with FBA with increasing levels of glucose. The figure shows the maximal rate of ATP that can be generated during light (upper panels) and dark (lower panels), in absence (left panels) and presence (right panels) of amino acids for the control model (orange squares) and for the retina-specific models. The control model contains two coupled Human1 models, while the retina-specific models consist of a RPE model coupled to a photoreceptor rod (‘rod’; green triangles) or cone (‘cone’; blue circles) model. The maximum permissible amino acid influx is determined by the ratio of amino acids to glucose in the bloodstream, normalized against the glucose exchange rate during light conditions or dark conditions (see methods), and was kept constant throughout the analyses. Furthermore, oxygen influx was fixed at 2.76 pmol·s^−1^·mm^−2^ in the light conditions and at 4.04 pmol·s^−1^·mm^−2^ in the dark conditions, and lactate efflux was fixed to 3.11 pmol·s^−1^·mm^−2^ in the light conditions and at 5.00 pmol·s^−1^·mm^−2^ in the dark conditions.


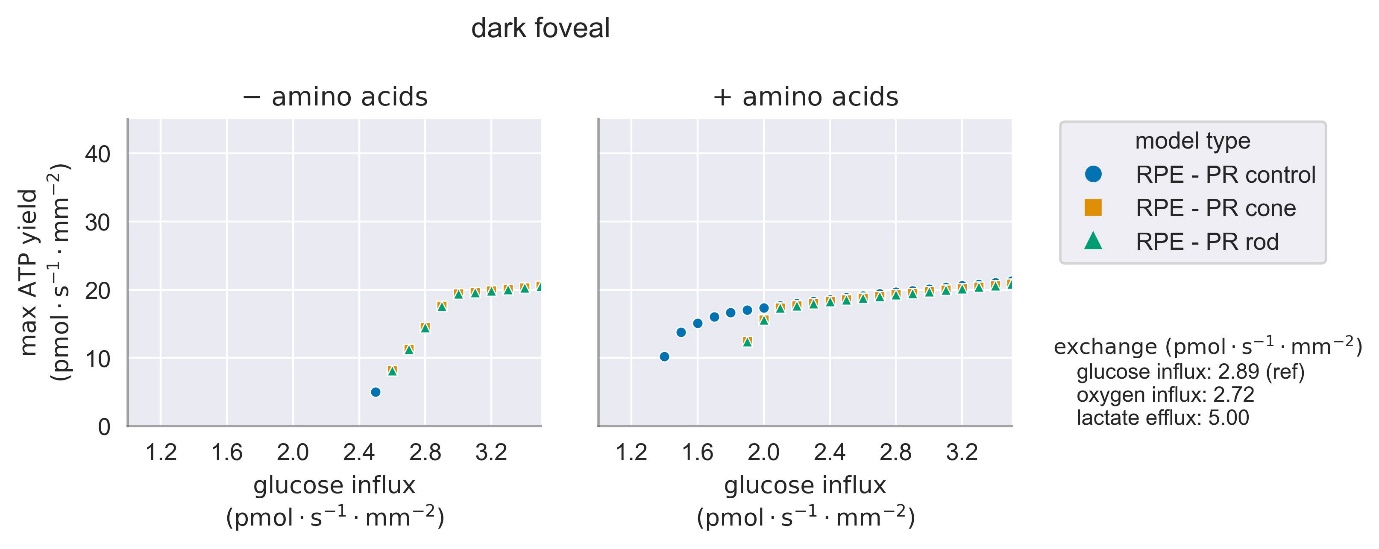

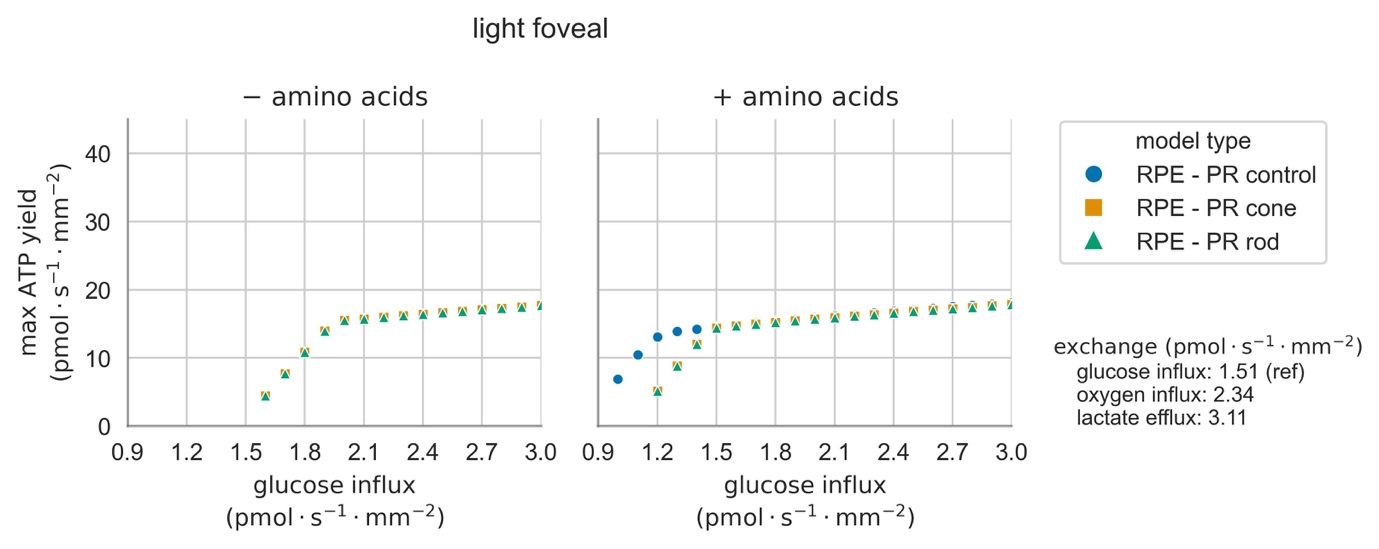

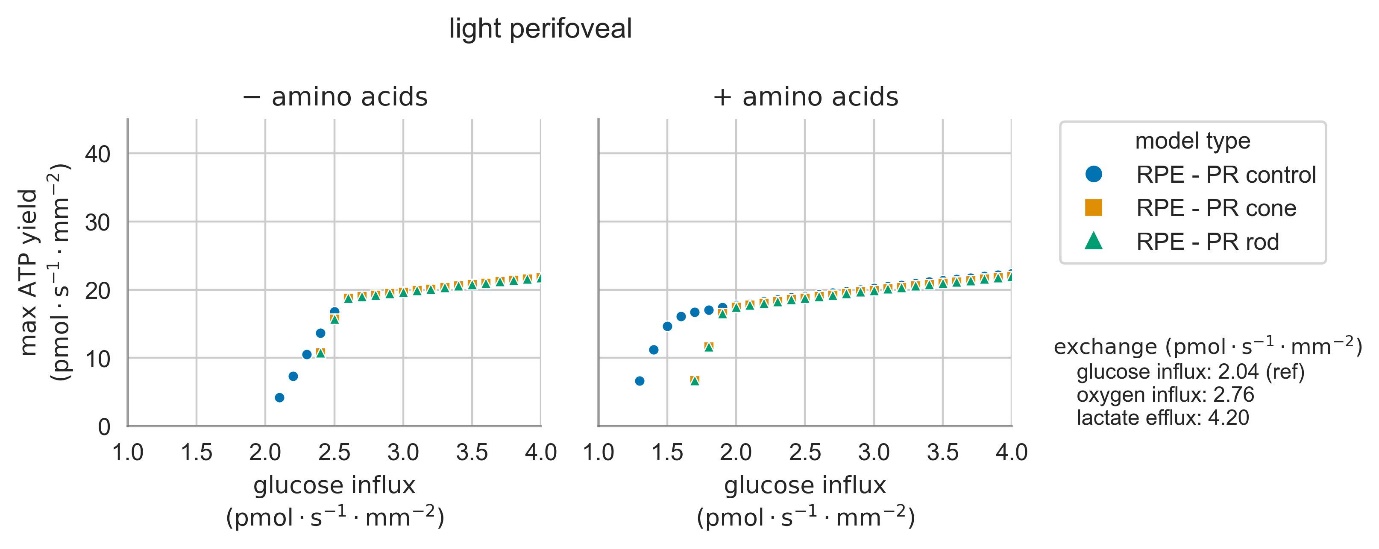

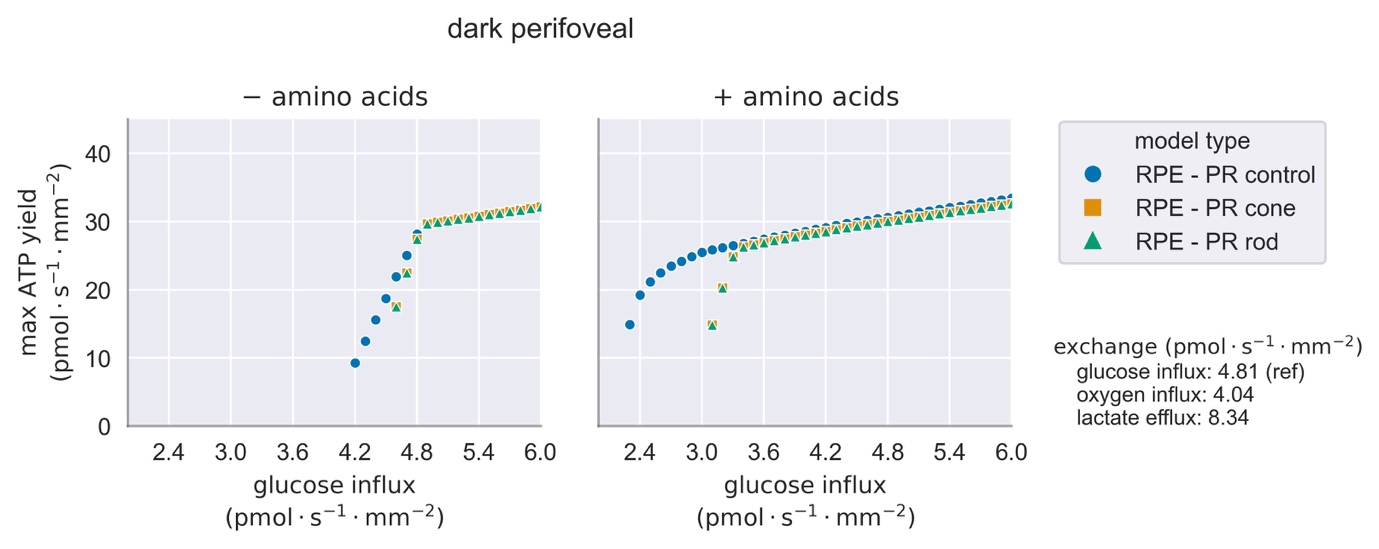


**Figure S4.** Maximal perifoveal ATP yield with increasing glucose influx. ATP hydrolysis was optimised with FBA with increasing levels of glucose. The figure shows the maximal rate of ATP that can be generated in pmol·s^−1^·mm^−2^ during light (upper panels) and dark (lower panels), in absence (left panels) and presence (right panels) of amino acids for the control model (orange squares) and for the retina-specific models. The control model contains two coupled Human1 models, while the retina-specific models consist of a RPE model coupled to a photoreceptor rod (‘rod’; green triangles) or cone (‘cone’; blue circles) model. The maximum permissible amino acid influx is determined by the ratio of amino acids to glucose in the bloodstream, normalized against the glucose exchange rate during light conditions or dark conditions (see methods), and was kept constant throughout the analyses. Furthermore, oxygen influx was fixed at 2.76 pmol·s^−1^·mm^−2^ in the light conditions and at 4.04 pmol·s^−1^·mm^−2^ in the dark conditions, and lactate efflux was fixed to 4.20 pmol·s^−1^·mm^−2^ in the light conditions and at 8.34 pmol·s^−1^·mm^−2^ in the dark conditions.

**Figure S5.** Maximal foveal ATP yield with increasing lactate efflux. ATP hydrolysis was optimised with FBA to investigate the effect of lactate efflux under different conditions on maximal ATP yield. The figure shows the maximal rate of ATP that can be generated in pmol·s^−1^·mm^−2^ during light (upper panels) and dark (lower panels), in absence (left panels) and presence (right panels) of amino acids for the control model (orange squares) and for the retina-specific models. The control model contains two coupled Human1 models, while the retina-specific models consist of a RPE model coupled to a photoreceptor rod (‘rod’; green triangles) or cone (‘cone’; blue circles) model. The maximum permissible amino acid influx is determined by the ratio of amino acids to glucose in the bloodstream, normalized against the glucose exchange rate during light conditions or dark conditions (see methods), and was kept constant throughout the analyses. Furthermore, oxygen influx was fixed at 2.34 pmol·s^−1^·mm^−2^ in the light conditions and at 2.72 pmol·s^−1^·mm^−2^ in the dark conditions, and glucose influx was fixed to 1.51 pmol·s^−1^·mm^−2^ in the light conditions and at 2.89 pmol·s^−1^·mm^−2^ in the dark conditions.


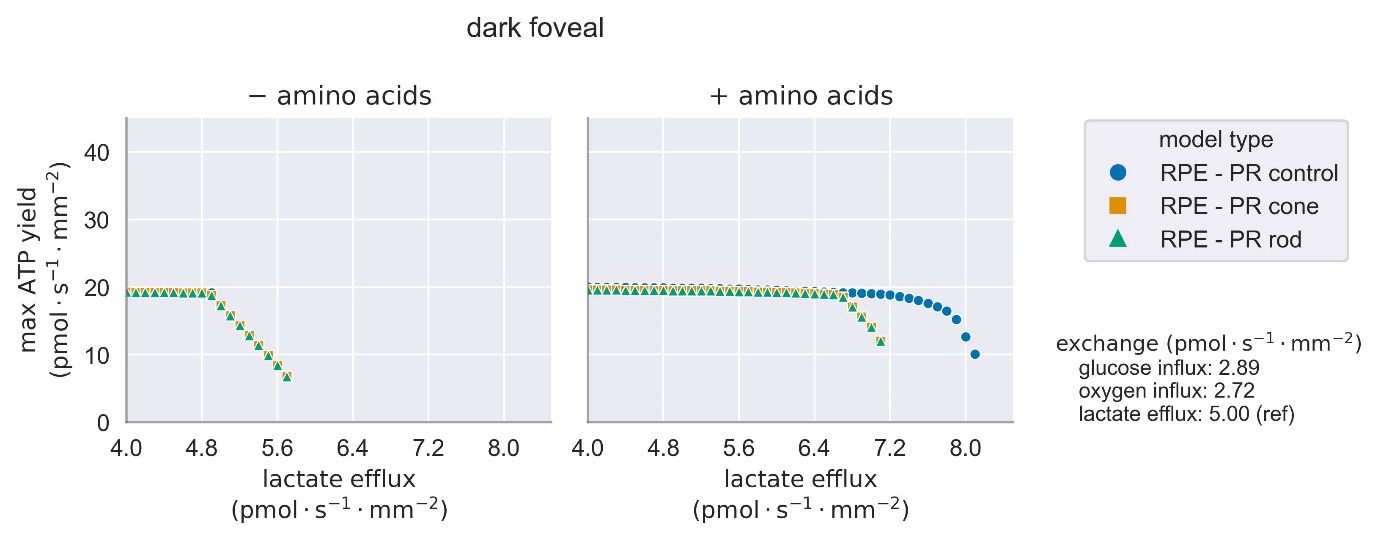

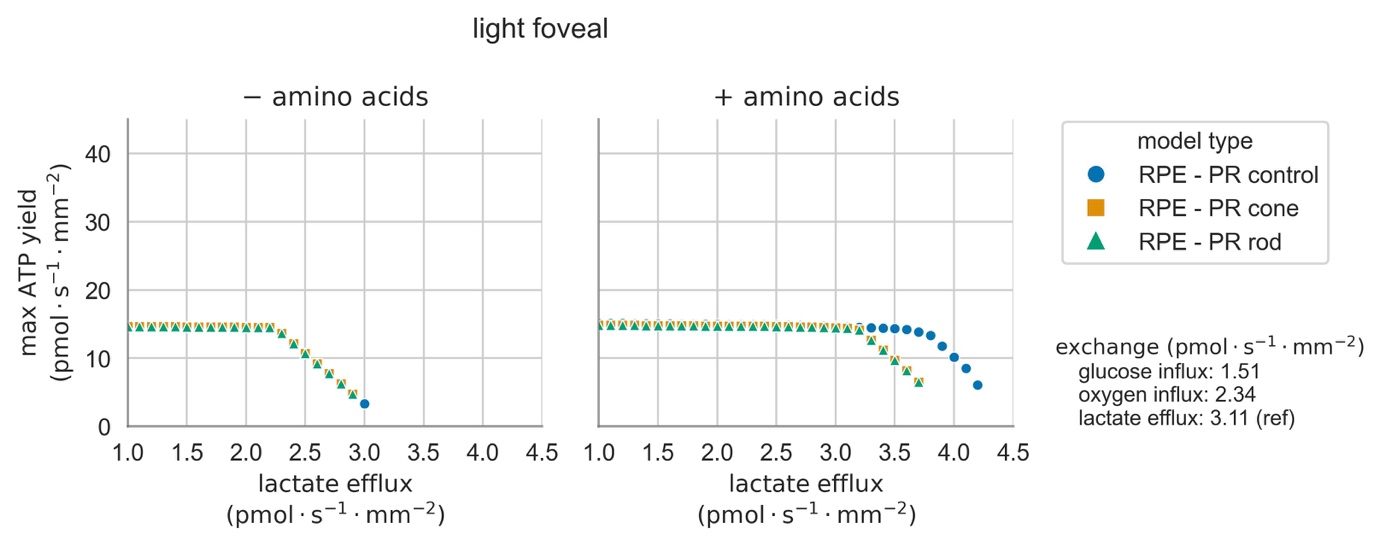


**Figure S6.** Maximal perifoveal ATP yield with increasing lactate efflux. ATP hydrolysis was optimised with FBA to investigate the effect of lactate efflux under different conditions on maximal ATP yield. The figure shows the maximal rate of ATP that can be generated in pmol·s^−1^·mm^−2^ during light (upper panels) and dark (lower panels), in absence (left panels) and presence (right panels) of amino acids for the control model (orange squares) and for the retina-specific models. The control model contains two coupled Human1 models, while the retina-specific models consist of a RPE model coupled to a photoreceptor rod (‘rod’; green triangles) or cone (‘cone’; blue circles) model. The maximum permissible amino acid influx is determined by the ratio of amino acids to glucose in the bloodstream, normalized against the glucose exchange rate during light conditions or dark conditions (see methods), and was kept constant throughout the analyses. Furthermore, oxygen influx was fixed at 2.76 pmol·s^−1^·mm^−2^ in the light conditions and at 4.04 pmol·s^−1^·mm^−2^ in the dark conditions, and glucose influx was fixed to 2.04 pmol·s^−1^·mm^−2^ in the light conditions and at 4.81 pmol·s^−1^·mm^−2^ in the dark conditions.


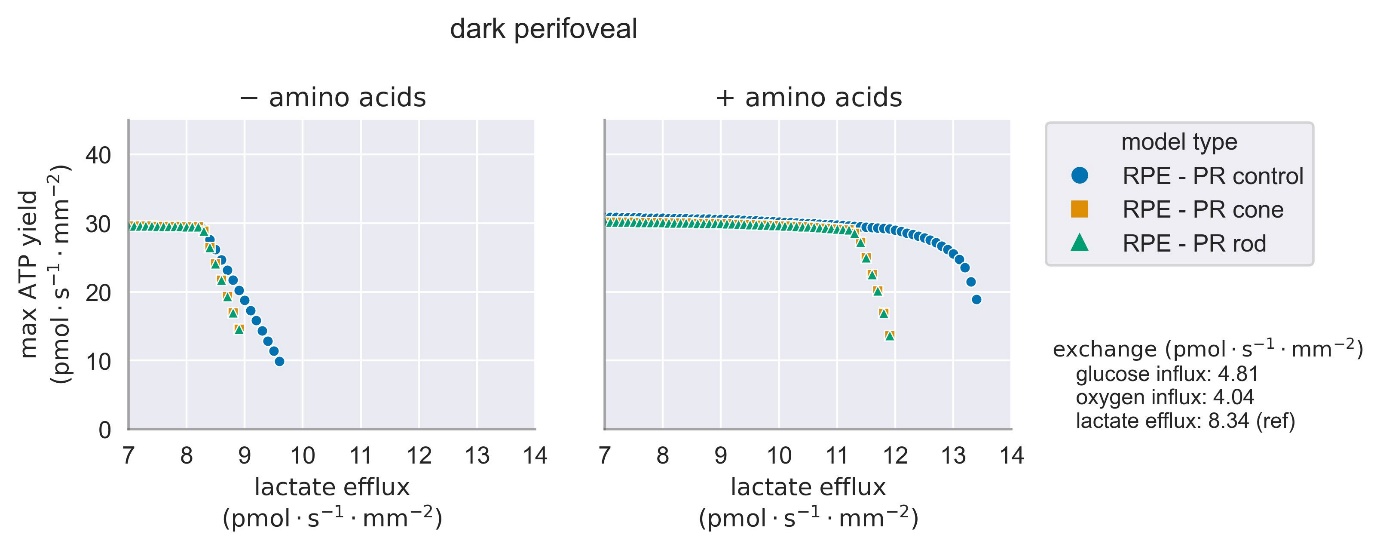

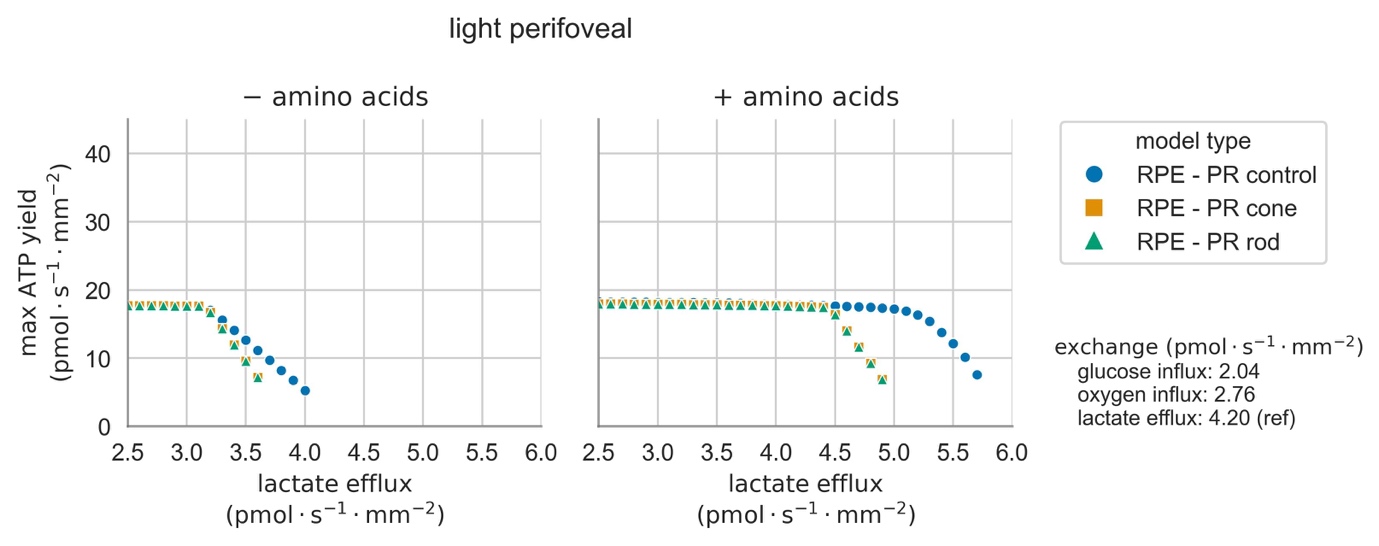


**Figure S7.** Maximal foveal ATP yield with increasing oxygen influx. ATP hydrolysis was optimised with FBA to investigate the effect of lactate efflux under different conditions on maximal ATP yield. The figure shows the maximal rate of ATP that can be generated in pmol·s^−1^·mm^−2^ during light (upper panels) and dark (lower panels), in absence (left panels) and presence (right panels) of amino acids for the control model (orange squares) and for the retina-specific models. The control model contains two coupled Human1 models, while the retina-specific models consist of a RPE model coupled to a photoreceptor rod (‘rod’; green triangles) or cone (‘cone’; blue circles) model. The maximum permissible amino acid influx is determined by the ratio of amino acids to glucose in the bloodstream, normalized against the glucose exchange rate during light conditions or dark conditions (see methods), and was kept constant throughout the analyses. Lactate and glucose fluxes are given to the right of the graphs. Analysis of the flux vectors at different levels of oxygen influx shows, at high oxygen fluxes, efflux of hydrogen peroxide which possibly explains the apparently paradoxical reduction in ATP yield at oxygen influx higher than that determined experimentally.


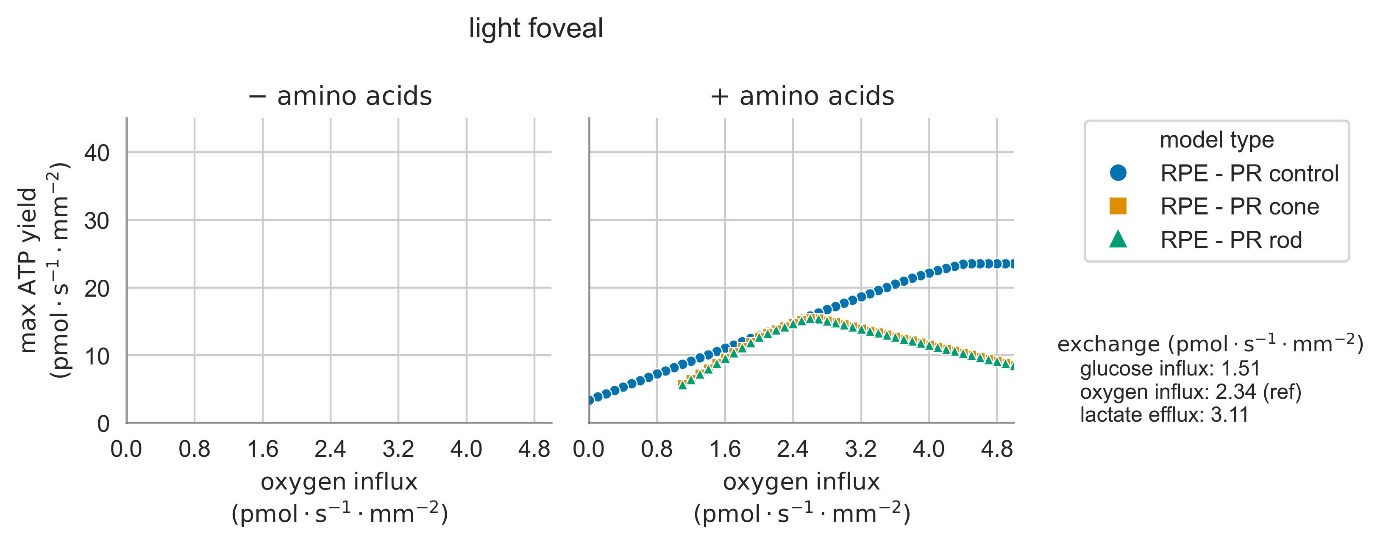

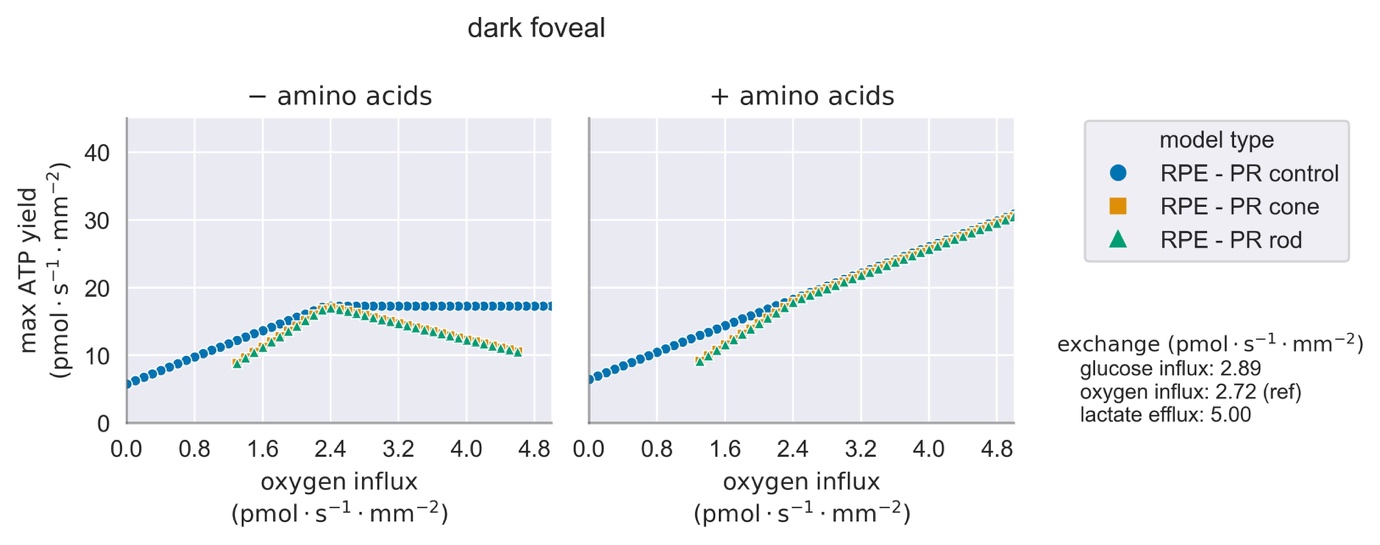

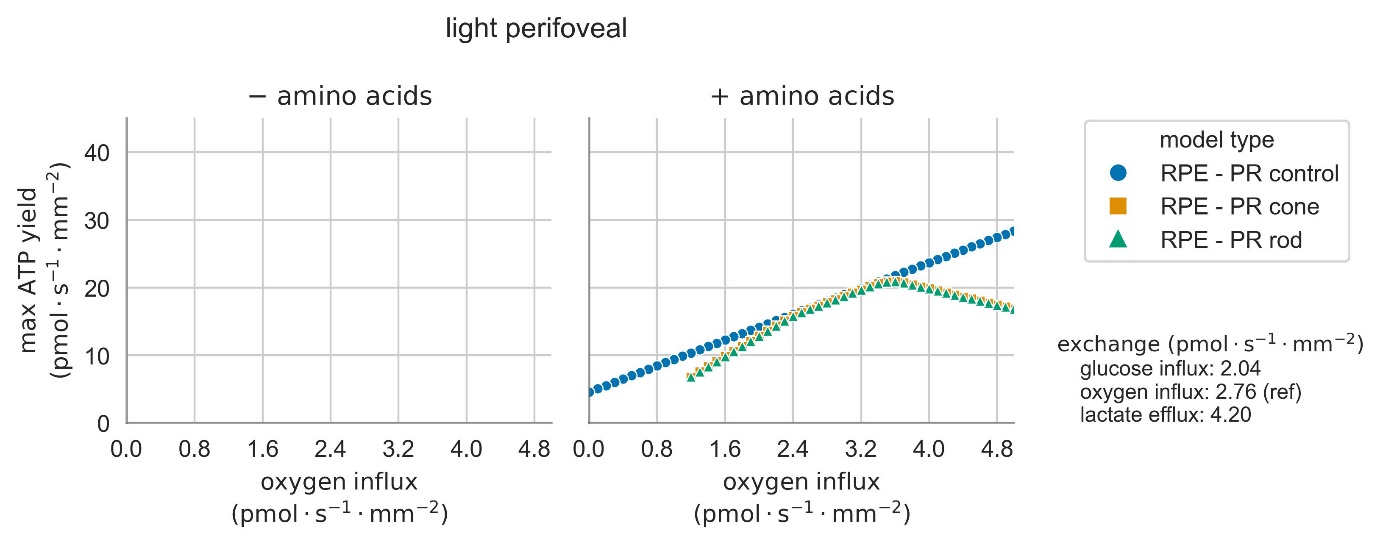

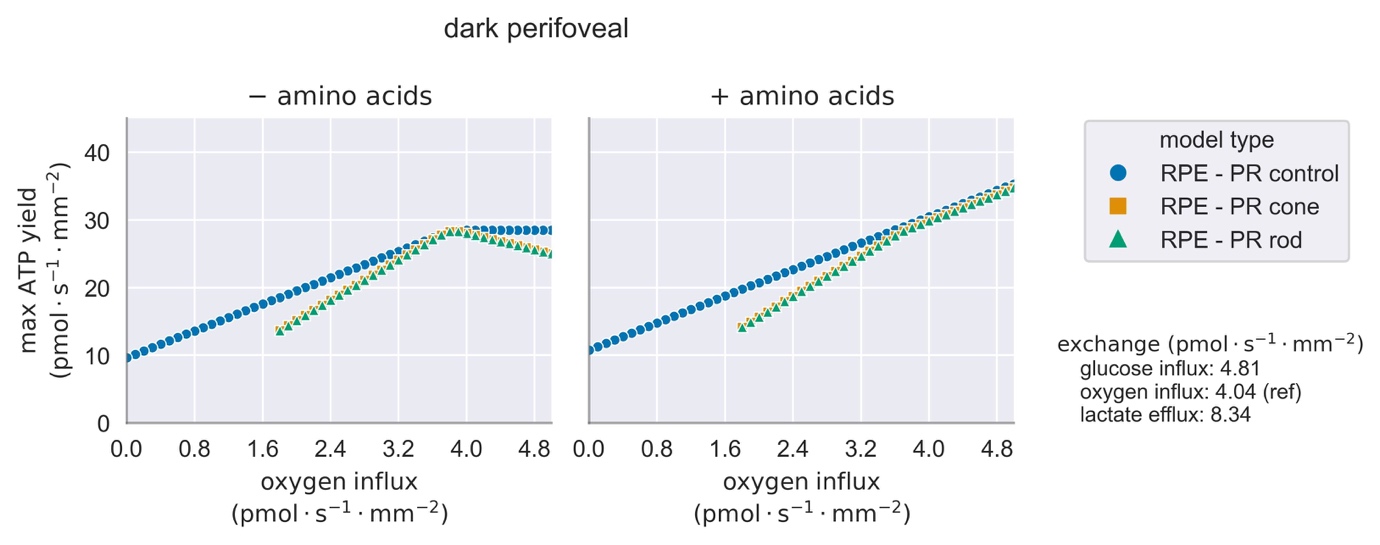


**Figure S8.** Maximal perifoveal ATP yield with increasing oxygen influx. ATP hydrolysis was optimised with FBA to investigate the effect of lactate efflux under different conditions on maximal ATP yield. The figure shows the maximal rate of ATP that can be generated in pmol·s^−1^·mm^−2^ during light (upper panels) and dark (lower panels), in absence (left panels) and presence (right panels) of amino acids for the control model (orange squares) and for the retina-specific models. The control model contains two coupled Human1 models, while the retina-specific models consist of a RPE model coupled to a photoreceptor rod (‘rod’; green triangles) or cone (‘cone’; blue circles) model. The maximum permissible amino acid influx is determined by the ratio of amino acids to glucose in the bloodstream, normalized against the glucose exchange rate during light conditions or dark conditions (see methods), and was kept constant throughout the analyses. Lactate and glucose fluxes are given to the right of the graphs.


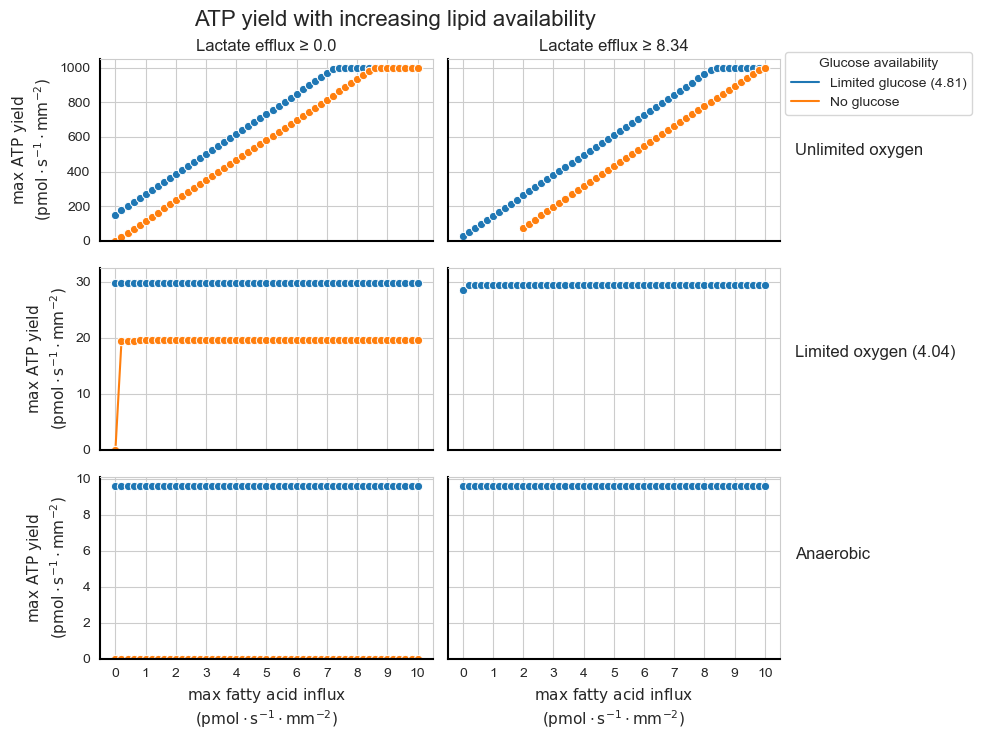
**Figure S9.** ATP yield with increasing lipid availability under different metabolic conditions. These graphs show the maximum ATP yield at varying availability of the fatty acid uptake pool (Human1 reaction ID: MAR13039) under conditions of unlimited and limited glucose and oxygen availability, as well as anaerobic conditions. The left panels show the yield with no imposed lactate efflux, while the right panels include a lactate efflux of at least 8.34 pmol·s^−1^·mm^−2^. The coloured lines represent different glucose availabilities: blue for limited glucose (4.81 pmol·s^−1^·mm^−2^) and orange for no glucose. The series of panels are further organized by oxygen availability: the top row under unlimited oxygen, the middle with oxygen influx constrained to 4.04 pmol·s^−1^·mm^−2^, and the bottom row under anaerobic conditions. The middle right panel contains the glucose, oxygen, and lactate exchange constraints in the perifoveal region during the dark.

**Figure S10.** Diagram showing compensatory age-related changes in rod diameter and choriocapillaris density. (**A**) In young adults there is a high density of photoreceptors (pale grey) and choriocapillaris profiles (red). Bruch’s membrane and retinal pigment epithelium are represented by the brown strip. (**B**) In elderly individuals the reduction in photoreceptor number leads to expansion of those remaining and a greater area for nutrient exchange so that is approximately compensated for by the reduction in choriocapillaris profiles. The capacity for exchange is represented with the blue rays.

| **Table S1.** Oxygen, glucose, and lactate exchange rates the cat choroid and eye as reported by Wang and colleagues (1997), converted to rates per unit area and ratios to oxygen exchange. | | | | |
| --- | --- | --- | --- | --- |
|  |  | nmol·min^−1^·retina^−1^ | pmol·s^−1^·mm^−2^ | ratio |
| light | oxygen | 166 | 2.80 | 1.00 |
|  | glucose | 123 | 2.07 | 0.74 |
|  | lactate | 253 | 4.27 | 1.52 |
| dark | oxygen | 198 | 3.34 | 1.00 |
|  | glucose | 236 | 3.98 | 1.19 |
|  | lactate | 409 | 6.90 | 2.07 |

| **Table S2.** Oxygen, glucose, and lactate exchange rates for exchange between the circulation and the RPE and the PR, and oxygen exchange between the RPE and PR in pmol·s^−1^·mm^−2^ | | | | | |
| --- | --- | --- | --- | --- | --- |
|  |  |  | RPE | PR | RPE --> PR |
| perifoveal | light | oxygen | 2.49 | 0.27 | 2.19 |
|  |  | glucose | 1.84 | 0.20 |  |
|  |  | lactate | 3.79 | 0.41 |  |
|  | dark | oxygen | 3.48 | 0.56 | 3.18 |
|  |  | glucose | 4.14 | 0.67 |  |
|  |  | lactate | 7.18 | 1.16 |  |
| foveal | light | oxygen | 2.34 |  | 2.04 |
|  |  | glucose | 1.51 |  |  |
|  |  | lactate | 3.11 |  |  |
|  | dark | oxygen | 2.72 |  | 2.42 |
|  |  | glucose | 2.89 |  |  |
|  |  | lactate | 5.00 |  |  |

| **Table S3.** Plasma concentrations of glucose and amino acids retrieved from the Human Metabolome Database (HMDB) | | | |
| --- | --- | --- | --- |
|  | HMDB symbol | Blood concentration (mM) | ratio to glucose |
| glucose | HMDB0000122 | 4.64 | 1.000 |
| glutamine | HMDB0000641 | 0.59 | 0.126 |
| alanine | HMDB0000161 | 0.42 | 0.091 |
| glycine | HMDB0000123 | 0.26 | 0.056 |
| lysine | HMDB0000182 | 0.24 | 0.051 |
| valine | HMDB0000883 | 0.23 | 0.050 |
| proline | HMDB0000162 | 0.19 | 0.041 |
| threonine | HMDB0000167 | 0.17 | 0.036 |
| serine | HMDB0000187 | 0.15 | 0.032 |
| leucine | HMDB0000687 | 0.12 | 0.026 |
| histidine | HMDB0000177 | 0.11 | 0.023 |
| arginine | HMDB0000517 | 0.08 | 0.017 |
| tyrosine | HMDB0000158 | 0.08 | 0.017 |
| isoleucine | HMDB0000172 | 0.07 | 0.014 |
| phenylalanine | HMDB0000159 | 0.06 | 0.013 |
| cysteine | HMDB0000574 | 0.05 | 0.011 |
| tryptophan | HMDB0000929 | 0.04 | 0.009 |
| glutamate | HMDB0000148 | 0.04 | 0.009 |
| asparagine | HMDB0000168 | 0.04 | 0.009 |
| methionine | HMDB0000696 | 0.03 | 0.005 |
| aspartate | HMDB0000191 | 0.02 | 0.003 |
| selenomethionine | HMDB0003966 | < 0.01 | < 0.001 |

| **Table S4**. Maximal ATP hydrolysis rate (pmol·s^−1^·mm^−2^) in absence (0 ×) and presence of different levels of amino acid influx (0.25 to 2 ×) | | | | | | |
| --- | --- | --- | --- | --- | --- | --- |
|  |  | *perifoveal* | |  | *foveal* | |
| model | × | light | dark |  | light | dark |
| RPE - PR control | 2 | 18.42 | 31.86 |  | 16.85 | 22.38 |
|  | 1 | 17.81 | 30.67 |  | 16.34 | 21.61 |
|  | 0.5 | 16.94 | 30.07 |  | 14.33 | 21.23 |
|  | 0.25 | 9.70 | 29.77 |  | 8.14 | 21.01 |
|  | 0 |  | 28.50 |  |  | 17.54 |
| RPE - PR rod | 2 | 17.93 | 30.62 |  | 16.46 | 21.59 |
|  | 1 | 17.59 | 30.05 |  | 16.14 | 21.22 |
|  | 0.5 | 8.25 | 29.76 |  | 6.04 | 21.04 |
|  | 0.25 |  | 29.62 |  |  | 20.92 |
|  | 0 |  | 27.90 |  |  | 15.50 |
| RPE - PR cone | 2 | 17.93 | 30.61 |  | 16.46 | 21.58 |
|  | 1 | 17.59 | 30.04 |  | 16.14 | 21.22 |
|  | 0.5 | 8.25 | 29.76 |  | 6.04 | 21.04 |
|  | 0.25 |  | 29.62 |  |  | 20.92 |
|  | 0 |  | 27.90 |  |  | 15.50 |

| **Table S5.** Glucose influx and lactate efflux rate thresholds required for feasible solutions (pmol·s^−1^·mm^−2^) given different blood exchange constraints with (+) and without (−) amino acids (aas), during light and dark conditons. | | | | | | | |
| --- | --- | --- | --- | --- | --- | --- | --- |
|  |  |  | *foveal* | |  | *perifoveal* | |
| model | light/dark | aas | glucose | lactate |  | glucose | lactate |
| RPE-PR control | dark | + | 1.4 | 8.1 |  | 2.3 | 13.4 |
|  |  | − | 2.5 | 5.7 |  | 4.2 | 9.6 |
|  | light | + | 1.0 | 4.2 |  | 1.3 | 5.7 |
|  |  | − | 1.6 | 3.0 |  | 2.1 | 4.0 |
| RPE-PRcone | dark | + | 1.9 | 7.1 |  | 3.1 | 11.9 |
|  |  | − | 2.6 | 5.7 |  | 4.6 | 8.9 |
|  | light | + | 1.2 | 3.7 |  | 1.7 | 4.9 |
|  |  | − | 1.6 | 2.9 |  | 2.4 | 3.6 |
| RPE-PRrod | dark | + | 1.9 | 7.1 |  | 3.1 | 11.9 |
|  |  | − | 2.6 | 5.7 |  | 4.6 | 8.9 |
|  | light | + | 1.2 | 3.7 |  | 1.7 | 4.9 |
|  |  | − | 1.6 | 2.9 |  | 2.4 | 3.6 |

| **Table S6.** Proportion of lipids in the fatty acid uptake pool | | | |
| --- | --- | --- | --- |
| Proportion | Lipid name | Proportion | Lipid name |
| 0.3837 | oleate | 0.0001 | cis-gondoic acid |
| 0.3015 | palmitate | 0.0001 | (11Z,14Z)-eicosadienoic acid |
| 0.1535 | linoleate | 0.0001 | cis-erucic acid |
| 0.0522 | palmitolate | 0.0001 | (11Z,14Z,17Z)-eicosatrienoic acid |
| 0.046 | stearate | 0.0001 | (13Z)-eicosenoic acid |
| 0.0338 | myristic acid | 0.0001 | (13Z)-octadecenoic acid |
| 0.0092 | linolenate | 0.0001 | (13Z,16Z)-docosadienoic acid |
| 0.0082 | arachidonate | 0.0001 | (4Z,7Z,10Z,13Z,16Z)-DPA |
| 0.0041 | DHA | 0.0001 | (6Z,9Z)-octadecadienoic acid |
| 0.002 | dihomo-gamma-linolenate | 0.0001 | (6Z,9Z,12Z,15Z,18Z)-TPA |
| 0.001 | EPA | 0.0001 | (6Z,9Z,12Z,15Z,18Z,21Z)-THA |
| 0.0001 | elaidate | 0.0001 | (7Z)-octadecenoic acid |
| 0.0001 | eicosanoate | 0.0001 | (7Z)-tetradecenoic acid |
| 0.0001 | DPA | 0.0001 | (9E)-tetradecenoic acid |
| 0.0001 | gamma-linolenate | 0.0001 | (9Z,12Z,15Z,18Z)-TTA |
| 0.0001 | henicosanoic acid | 0.0001 | (9Z,12Z,15Z,18Z,21Z)-TPA |
| 0.0001 | lauric acid | 0.0001 | 10,13,16,19-docosatetraenoic acid |
| 0.0001 | lignocerate | 0.0001 | 10,13,16-docosatriynoic acid |
| 0.0001 | (10Z)-heptadecenoic acid | 0.0001 | 12,15,18,21-tetracosatetraenoic acid |
| 0.0001 | margaric acid | 0.0001 | 13,16,19-docosatrienoic acid |
| 0.0001 | cis-vaccenic acid | 0.0001 | 7-palmitoleic acid |
| 0.0001 | nervonic acid | 0.0001 | 8,11-eicosadienoic acid |
| 0.0001 | nonadecylic acid | 0.0001 | 9-eicosenoic acid |
| 0.0001 | omega-3-arachidonic acid | 0.0001 | 9-heptadecylenic acid |
| 0.0001 | pentadecylic acid | 0.0001 | adrenic acid |
| 0.0001 | physeteric acid | 0.0001 | behenic acid |
| 0.0001 | stearidonic acid | 0.0001 | cerotic acid |
| 0.0001 | tricosanoic acid | 0.0001 | cis-cetoleic acid |
| 0.0001 | tridecylic acid | 0.0001 | ximenic acid |
| 0.0001 | mead acid |  |  |
