# Supplementary material for: “Energetics of the outer retina I: Estimates of nutrient exchange and ATP generation”
Source: PLoS One. 2024 Dec 31;19(12):e0312260. doi: 10.1371/journal.pone.0312260 (PMC11687866; doi:10.1371/journal.pone.0312260)
Supplement: Attachment — Submitted filename: Response to Reviewers.docx [file pone.0312260.s002.docx]

**Response to Reviews Comments**

**Reviewer #1:** In this study the authors aimed to explore how in healthy aging there are concurrent shifts in both supply and demand, which may be critical in maintenance of homeostasis of the aging eye.

*Comment 1: “The study heavily relies on computational simulations and extrapolations from animal data due to the challenges of directly measuring nutrient fluxes and metabolic activities in the human retina. While efforts were made to validate through available experimental data (such as oxygen tension profiles), direct validation of the entire metabolic model in vivo remains challenging.”*

Reply 1: This is an important point. Part of the classical cycle of computational modelling involves validation of model predictions that were not ‘baked in’ to the original model (see Figure 1 in ^1^). Unfortunately, as we mention (lines 88–95), many of the key parameters of interest in relation to human outer retinal metabolism in vivo are simply inaccessible to current experimental methods. So, in this instance we are offering plausible hypotheses as to what might be going on rather than providing definitive proof. The hope is that this work will at least in part help provoke development of technologies that can validate our findings.

**We have strengthened the description of study limitations in the discussion (lines 598-602). “**Model validation is an important challenge but, as here, the direct measurements that would be required for validation are often not possible with current methodologies and the modelling can be seen as hypothesis-generating or as offering feasible solutions but not direct demonstration of what is taking place.”

*Comment 2: “The study provides insights into static metabolic states (light vs. dark conditions), but retinal metabolism is dynamic and can vary under different physiological and pathological states (e.g., aging, disease). The model's ability to capture these dynamic changes is limited by current data and assumptions.”*

Reply 2: We have indeed only modelled static steady states. Dynamic changes, which are of great interest, require a fully parametrised kinetic model. This is a major undertaking and we are working on this but realistically we are 1 – 2 years away. Other groups may be closer. One of the benefits of the current study is that it provides steady-state points against which to compare a dynamic model.

**We have articulated this thinking in the revised discussion and provided a reference to a publication that discusses metabolic dynamics of the retina (lines 595-8). “**Flux balance analysis, as employed here, only captures steady-state phenomena, whereas in reality, the retina is a dynamic system responding swiftly to, for instance, changes in illumination, which will bring specific metabolic challenges^2^, that are not addressed by the current approach.”

*Comment 3: “While the study lays a foundation for understanding normal retinal bioenergetics, translating these findings to clinical applications (e.g., understanding retinal diseases or therapeutic strategies) requires further validation and adaptation to human clinical context”*

Reply 3: We completely agree. The current work is the first step towards gaining deeper understanding of diseases and therapeutics. Many further steps will be required to achieve the clinical impact we ultimately seek to achieve.

**We have included this towards the end of the revised discussion (lines 602-5). “**The ultimate aim of this work is to be able to predict evolution of changes with age and disease, but this is well beyond the scope of this current study. Validation of such a model would itself require clinical determination of its predictive capability. “

*Comment 4: “The study's exploration of age-related changes in retinal bioenergetics highlights complexities in metabolic interactions between different retinal layers and their vascular supply. However, understanding how these interactions evolve over time, especially in the context of disease-related metabolic shifts, warrants further investigation beyond the study's current scope.”*

Reply 4: We agree.

**A statement to this effect has been incorporated into the revised discussion (lines 602-5). “**The ultimate aim of this work is to be able to predict evolution of changes with age and disease, but this is well beyond the scope of this current study. Validation of such a model would itself require clinical determination of its predictive capability. “

**Reviewer #2:** Strengths:
*Comment 5: “The authors used a genome-scale metabolic computational program to mathematically model the production of metabolic energy in rod and cone photoreceptors and in the retinal pigment epithelium. The GEM modeling program (Human1) and constraint-based models are established and widely used so I think this now is considered by many to be an important way to initiate a comprehensive overview of metabolism in many types of cells. The authors also use data from oxygen consumption measurements to constrain their analyses and measurements of glucose and lactate in the arteries and veins leading to and from a mammalian retina. The major strength of this report is that it is a pioneering study of this type of computational analysis of the retina and RPE. It is important because it will serve as a starting point for future analyses that steadily improve on the modeling as more and more data become available. Analyses of what happens when expression of specific enzymes are blocked and comparison with data from actual metabolic analyses of animals in which those genes are inactivated will be very useful in future studies. Overall, the authors describe their strategies and findings clearly in this manuscript. The analyses used single-cell RNA sequencing data that I think most investigators consider to be reliable.”*

Reply 5: We are grateful for the encouragement! We also see this work as a starting point from which we and other groups can build models of increasing biological fidelity.

**We have emphasised this in the revised discussion (lines 593-5). “**There are many additional limitations to this study and indeed, we consider it to be a first step towards a high fidelity, integrated description of the bioenergetics of the human retina and associated tissues.”

*Weaknesses:
Comment 6: “I think it would be surprising that a model based on transcript abundances could accurately predict the actual abundances of the relevant proteins and their activities. It may be possible for the authors to address this. There is published data that might provide a way to test the validity of the assumption that transcript abundance is proportional to protein abundance and activity. There is a published analysis of expression of phosphoinositide kinase and phosphatase proteins in RPE and photoreceptor cells that was reported by Rajala et al. (PMID 37007713 and 35427794). Those papers quantified the expression of PI kinases and phosphatases in specific cell types by isolating ribosomes specifically either from RPE cells, rods, cones or other cells. It would be useful for the authors (Prins et al.) to compare the actual levels of expression of those proteins (PI kinases and phosphatases) in rods, cones and RPE cells reported in those studies with the levels of RNA transcripts reported from the Iowa RNA seq studies that they used in their GEM analyses. If transcript and protein abundances are in good agreement with each other then the authors could use that to strengthen the interpretation of their analyses. If they do not agree then the authors should still report their findings but they should include for their readers a discussion of the extent to which quantifying transcripts rather than the actual amounts of enzymes and their activities adds uncertainty to the interpretation.”*

Reply 6: We are grateful for being directed to the interesting PI kinase and phosphatase papers, which will doubtless inform our future studies. From our reading, the methodology used involves assaying actively transcribed mRNA and therefore the data do not represent actual protein abundances).

There is certainly an important issue when seeking to extrapolate from mRNA to protein abundancies. The correlation is often poor. As others have pointed out^3^ , while there is an abundance of RNA-seq and proteomics data available, the majority of these datasets are unpaired with RNA-seq and proteomics conducted under different conditions. Given the varying dynamic ranges and technical biases across different mRNA and protein assays, merging of various datasets might potentially magnify the divergence between mRNA and protein levels.

There have been several approaches to address this problem in GEM studies. The CORDA method used in this work, doesn’t hinge on precise expression levels. Rather, it categorizes expression data into five distinct levels: unknown, not detected, low confidence, medium confidence, and high confidence. The expression confidence levels are mapped on the reactions after which the algorithm includes all high confidence reactions and maximizes the inclusion of medium confidence reactions, while minimizing the incorporation of non-present reactions. This approach determines whether or not reactions can occur, rather than setting the precise magnitude boundaries of the reactions. This strategy mitigates the impact of the uncertainty between mRNA and protein levels. While the correlation between mRNA and protein levels may be weak, the CORDA method allows for a reasonable representation of the system’s state.

We completely agree that the use of protein abundances would be optimal and hope that in future studies it will be possible to integrate proteomic and immunohistochemical data into network definition and flux constraints or rates.

**We have amplified the discussion relating to the challenges of using mRNA as opposed to protein abundances and given additional references (lines 491-500). “**The correlation between mRNA and protein abundance is a contentious issue, with studies showing varying degrees of correlation^4^. This challenge exacerbated by the fact that most RNA-seq and proteomics datasets are unpaired and conducted under different conditions^3^ In this work, the CORDA method was employed to create RPE, rod- and cone-specific models, mitigating some of these uncertainties. Rather than using precise RNA levels, RNA sequencing data was categorised into distinct confidence levels to determine whether or not specific reactions should be included in the network. When it becomes available, integration of proteomic data to refine network reaction boundaries has the potential to improve the biological fidelity of the models.”

Comment from companion paper Kiel et al

Comment 32: “*3. lines 347-353. It would be helpful to discuss the importance of mitochondrial coupling. Photoreceptor mitochondria may be more uncoupled than in most other types of cells. The authors cite reference 46 (in paper 2), which is based on measurements of mitochondria from cultured cells and from mitoplast preparation. The properties of mitochondria can vary in the degree to which they are uncoupled and photoreceptor mitochondria may be particularly unique in this way. This should be discussed.*”

Response 32: The issue of mitochondrial uncoupling is important and we completely agree that photoreceptor mitochondria may be unique! Oxygen consumption rate doesn’t increase greatly following uncoupling ^5^, which could reflect basal activity being very high, as in a cardiomyocyte ^6^. But we agree that the limited drop in OCR with oligomycin may well reflect a significant degree of basal uncoupling. Nevertheless, curiously, and perhaps reflecting that the effect isn’t massive, inhibiting oxidative phosphorylation results in a significant drop in ATP production for the retina (see Fig 1 in the Cepko group’s paper ^7^). It’s not clear how much of this drop relates to photoreceptors and how much to inner retina.

**We have added a comment about this (lines 575-9), emphasising that uncoupling may explain why our estimates of ATP production are higher than our estimates of demand. We have also added references to Dr Hurley’s work on this topic^8, 9^, to illustrate the point. “**FBA optimises ATP yield and assumes the system is 100% efficient, which is probably not the case. Indeed, there are data suggesting that photoreceptor mitochondria are uncoupled to an unusual degree, which would reduce ATP production whilst oxygen fluxes were maintained^8, 9^. “

**References**

1.	Roberts PA, Gaffney EA, Luthert PJ, et al. Mathematical and computational models of the retina in health, development and disease. *Prog Retin Eye Res* 2016; 53: 48-69. 20160407. DOI: 10.1016/j.preteyeres.2016.04.001.

2.	Chen Y, Zizmare L, Calbiague V, et al. Retinal metabolism displays evidence for uncoupling of glycolysis and oxidative phosphorylation via Cori-, Cahill-, and mini-Krebs-cycle. *Elife* 2024; 12 20240513. DOI: 10.7554/eLife.91141.

3.	Prabahar A, Zamora R, Barclay D, et al. Unraveling the complex relationship between mRNA and protein abundances: a machine learning-based approach for imputing protein levels from RNA-seq data. *NAR Genom Bioinform* 2024; 6: lqae019. 20240210. DOI: 10.1093/nargab/lqae019.

4.	Munro V, Kelly V, Messner CB, et al. Cellular control of protein levels: A systems biology perspective. *Proteomics* 2024; 24: e2200220. 20231127. DOI: 10.1002/pmic.202200220.

5.	Kooragayala K, Gotoh N, Cogliati T, et al. Quantification of Oxygen Consumption in Retina Ex Vivo Demonstrates Limited Reserve Capacity of Photoreceptor Mitochondria. *Invest Ophthalmol Vis Sci* 2015; 56: 8428-8436. 2016/01/10. DOI: 10.1167/iovs.15-17901.

6.	Mootha VK, Arai AE and Balaban RS. Maximum oxidative phosphorylation capacity of the mammalian heart. *Am J Physiol* 1997; 272: H769-775. DOI: 10.1152/ajpheart.1997.272.2.H769.

7.	Chinchore Y, Begaj T, Wu D, et al. Glycolytic reliance promotes anabolism in photoreceptors. *Elife* 2017; 6 20170609. DOI: 10.7554/eLife.25946.

8.	Hass DT, Bisbach CM, Sadilek M, et al. Aerobic Glycolysis in Photoreceptors Supports Energy Demand in the Absence of Mitochondrial Coupling. *Adv Exp Med Biol* 2023; 1415: 435-441. DOI: 10.1007/978-3-031-27681-1_64.

9.	Du J, Rountree A, Cleghorn WM, et al. Phototransduction Influences Metabolic Flux and Nucleotide Metabolism in Mouse Retina. *J Biol Chem* 2016; 291: 4698-4710. 20151216. DOI: 10.1074/jbc.M115.698985.
